# Hyperbaric oxygen treatment for long coronavirus disease-19: a case report

**DOI:** 10.1186/s13256-022-03287-w

**Published:** 2022-02-15

**Authors:** Aisha M. Bhaiyat, Efrat Sasson, Zemer Wang, Sherif Khairy, Mouzayan Ginzarly, Umair Qureshi, Moin Fikree, Shai Efrati

**Affiliations:** 1 Aviv Clinics, Jumeirah Lake Towers, Dubai, United Arab Emirates; 2 Aviv Scientific Ltd, 7 Mezada Street, Bnei Brak, Israel; 3 Rashid Hospital Trauma Center, Dubai, United Arab Emirates; 4 Sagol Center for Hyperbaric Medicine and Research, Shamir Medical Center, Israel Sackler School of Medicine and Sagol School of Neuroscience, Tel-Aviv University, Tel Aviv, Israel

**Keywords:** COVID-19, SARS-CoV-2, Case report, Hyperbaric oxygen therapy, HBOT, Long COVID

## Abstract

**Background:**

The coronavirus disease 2019 pandemic has resulted in a growing population of individuals who experience a wide range of persistent symptoms referred to as “long COVID.” Symptoms include neurocognitive impairment and fatigue. Two potential mechanisms could be responsible for these long-term unremitting symptoms: hypercoagulability, which increases the risk of blood vessel occlusion, and an uncontrolled continuous inflammatory response. Currently, no known treatment is available for long COVID. One of the options to reverse hypoxia, reduce neuroinflammation, and induce neuroplasticity is hyperbaric oxygen therapy. In this article, we present the first case report of a previously healthy athletic individual who suffered from long COVID syndrome treated successfully with hyperbaric oxygen therapy.

**Case presentation:**

A previously healthy 55-year-old Caucasian man presented 3 months after severe coronavirus disease 2019 infection with long COVID syndrome. His symptoms included a decline in memory, multitasking abilities, energy, breathing, and physical fitness. After evaluation that included brain perfusion magnetic resonance imaging, diffusion tensor imaging, computerized cognitive tests, and cardiopulmonary test, he was treated with hyperbaric oxygen therapy. Each session included exposure to 90 minutes of 100% oxygen at 2 atmosphere absolute pressure with 5-minute air breaks every 20 minutes for 60 sessions, 5 days per week. Evaluation after completing the treatment showed significant improvements in brain perfusion and microstructure by magnetic resonance imaging and significant improvement in memory with the most dominant effect being on nonverbal memory, executive functions, attention, information procession speed, cognitive flexibility, and multitasking. The improved cognitive functions correlated with the increased cerebral blood flow in brain regions as measured by perfusion magnetic resonance imaging. With regard to physical capacity, there was a 34% increase in the maximum rate of oxygen consumed during exercise and a 44% improvement in forced vital capacity. The improved physical measurements correlated with the regain of his pre-COVID physical capacity.

**Conclusions:**

We report the first case of successfully treated long COVID symptoms with hyperbaric oxygen therapy with improvements in cognition and cardiopulmonary function. The beneficial effects of hyperbaric oxygen shed additional light on the pathophysiology of long COVID. As this is a single case report, further prospective randomized control studies are needed.

## Background

The coronavirus disease 2019 (COVID-19) pandemic has resulted in a growing population of individuals who experience a wide range of long-lasting symptoms after recovery from the acute illness, referred to by several terms, including “post-COVID conditions” and “long COVID.” The five most common symptoms recognized post-COVID are fatigue (58%), headache (44%), cognitive impairment (27%), hair loss (25%), and dyspnea (24%) [1].

Two main biological sequelae of COVID-19 play roles in the pathogenesis of long COVID. The first is hypercoagulability state characterized by increased risk of small- and large-vessel occlusion [2]. The second is an uncontrolled continuous inflammatory response [3]. Microinfarcts and neuroinflammation are important causes of brain hypoxia and can be responsible for the chronic unremitting neurocognitive decline in patients with long COVID [4]. One of the options to reverse hypoxia, reduce neuroinflammation, and induce neuroplasticity is hyperbaric oxygen therapy (HBOT) [5]. In this article, we present the first case report of previously healthy, athletic individual who suffered from long-standing post-COVID syndrome treated successfully with HBOT.

## Case presentation

A 55-year-old previously healthy Caucasian man suffering from persistent unremitting symptoms of long COVID attended our clinic for evaluation. The clinical presentation included memory problems, worsening of multitasking abilities, fatigue, low energy, breathlessness, and reduced physical fitness, which all started after acute SARS-CoV-2 infection diagnosed 3 months before. He initially developed high-grade fever without chest pain, cough, or shortness of breath, on 21 January 2021. He was admitted to hospital because of dehydration on 30 January 2021 and was diagnosed with COVID-19 by reverse-transcription polymerase chain reaction (RT-PCR). During the hospital stay, he developed acute respiratory syndrome due to pneumonitis and required supportive treatment with high-flow oxygen for 1 week. He was discharged from hospital on 16 February 2021. At discharge, he was stable with normal oxygen and no neurological deficiencies were noted on physical examination. In addition, 6 weeks after being diagnosed with COVID-19, he developed a pulmonary embolus and was treated with rivaroxaban. Prior to the SARS-CoV-2 infection, he had been a healthy, high-functioning, and athletic individual.

The baseline evaluation done at our clinic, 3 months after the acute infection, included brain magnetic resonance imaging (MRI) with perfusion and diffusion tensor imaging (DTI), computerized neurocognitive evaluation, cardiopulmonary exercise test (CPET), and pulmonary function tests.

At baseline, the patient complained of shortness of breath with exercise as well as difficulties with memory and multitasking that started after his COVID-19 illness. Physical and neurological examination was normal. Brain MRI evaluation demonstrated reduced perfusion that correlated with the cognitive decline as detailed below. He was referred to hyperbaric oxygen therapy (HBOT) that included 60 sessions, 5 days per week. Each session included exposure to 90 minutes of 100% oxygen at 2 atmosphere absolute with 5-minute air breaks every 20 minutes.

The patient started his first HBOT on 19 April 2021 and finished on 15 July 2021 without any significant side effects. After the first five sessions, he reported that his breathing had started to improve and that he no longer had muscle aches after exercise. After 15 sessions, he noted less fatigue and an improvement in his previous low energy. After 20 sessions, he noticed that his breathing and exercise capacity had returned to his capacity pre-SARS-CoV-2 infection, returning to running mountain trails. Additionally, he noted that his memory and multitasking ability returned to his pre-COVID-19 levels.

**Fig. 1:**
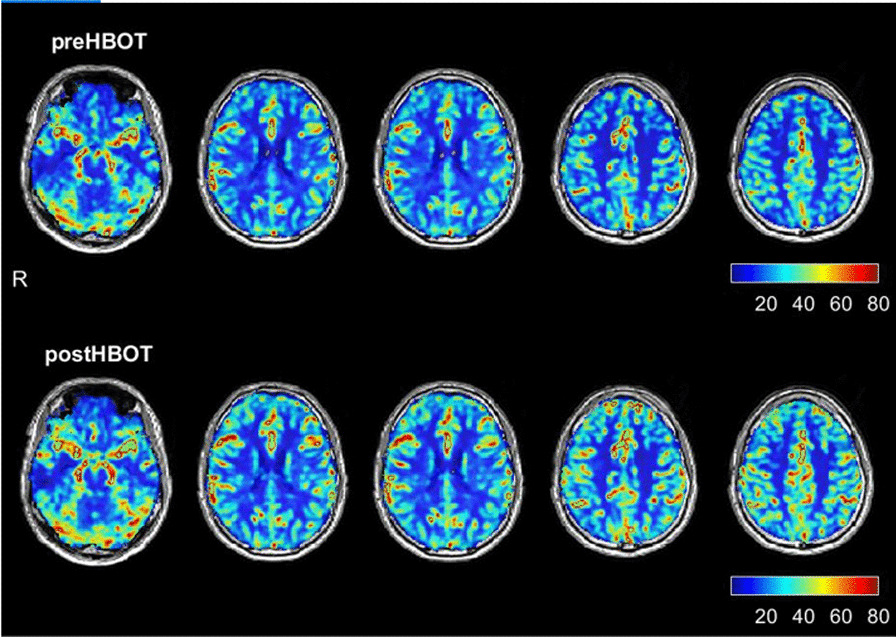
Brain perfusion magnetic resonance imaging before and after hyperbaric oxygen therapy. The upper row represents brain perfusion 3 months after the acute infection, before hyperbaric oxygen therapy. The lower row represents the perfusion magnetic resonance imaging done after completing the hyperbaric oxygen therapy protocol

The baseline brain MRI, prior to the HBOT, showed two small foci of signal alterations in the right and left parietal regions suggestive of early small-vessel disease. In addition, there was a global decrease in the brain perfusion. As detailed in Fig. 1 and Table 1, re-evaluation after HBOT (done 4 weeks after the last HBOT to avoid any potential intermediate effect) revealed a significant increase in brain perfusion. Tables 2 and 3 present the improvements in the brain microstructure as demonstrated by MRI–DTI.

**Table 1:** Brain blood flow changes before and after hyperbaric oxygen therapy

Brain region	Pre-HBOT	Post-HBOT	Change in %
White matter right (R)	19.43	22.89	17.80
White matter left (L)	19.17	22.23	16
Gray matter R	32.34	38.6	19.40
Gray matter L	33.3	38.91	16.80
Primary gustatory cortex R	34.22	47.43	38.60
Lateral postcentral gyrus R	32.08	42.79	33.40
Superior temporal gyrus R	38.04	50.65	33.10
Supramarginal gyrus R	36.37	46.39	27.60
Anterior cingulate cortex L	40.16	50.61	26
Inferior frontal gyrus L	39.47	49.6	25.70
Inferior frontal gyrus (Broca’s area) R	37.55	46.81	24.70
Medial frontal gyrus R	29.57	36.67	24

**Table 2:** Magnetic resonance imaging–diffusion tensor imaging fractional anisotropy changes before and after hyperbaric oxygen therapy

Brain region	Pre-HBOT	Post-HBOT	Change in %
Superior fronto-occipital fasciculus L	0.44	0.48	7.52
Cingulum (hippocampus) R	0.24	0.26	7.46
Superior corona radiata L	0.39	0.42	5.63
Body of corpus callosum	0.43	0.45	5.39
Cingulum (hippocampus) L	0.23	0.24	4.59
Corticospinal tract L	0.37	0.38	3.49
External capsule L	0.36	0.38	3.23
Superior corona radiata R	0.43	0.44	3.21

Fractional anisotropy (FA) is a measure used to evaluate white matter fiber integrity, directionality, and order. A higher value of FA indicates better fiber organization.

*DTI* diffusion tensor imaging

**Table 3:** Magnetic resonance imaging–diffusion tensor imaging mean diffusivity changes before and after hyperbaric oxygen therapy

Brain region	Pre-HBOT	Post-HBOT	Change in %
Medial lemniscus R	1.3	1.24	4.72
Superior longitudinal fasciculus L	0.76	0.73	4.61
Medial lemniscus L	1.23	1.18	4.34
Superior corona radiata L	0.77	0.74	3.18
Superior fronto-occipital fasciculus L	0.75	0.72	3.14
Sagittal stratum L	0.83	0.81	2.51
Pontine crossing tract	0.76	0.75	2.35
Fornix L	1.01	0.99	2.06

Mean diffusivity (MD) is a measure used to evaluate white matter fiber density. A lower value of MD indicates a higher density.

*DTI* diffusion tensor imaging

Neurocognitive assessment was done using NeuroTrax full computerized testing battery to measure different aspects of brain function, such as memory, information processing speed, attention, and executive function, was done before and after HBOT. The post-HBOT neurocognitive testing showed significant improvement in global memory with the most dominant effect being on nonverbal memory, executive functions, attention, information procession speed, cognitive flexibility, and multitasking. Table 4 summarizes the pre- and post-HBOT scores in the different cognitive domains.

**Table 4:** Cognitive scores before and after hyperbaric oxygen therapy

Neurotrax	Pre-HBOT	Post-HBOT	Change in %
Global cognitive score	93.3	99.4	6.5
Memory	98.8	105.8	7.1
Nonverbal memory	96.2	114	18.5
Delayed nonverbal memory	105.6	113.6	7.6
Verbal memory	92.1	94.5	2.6
Delayed verbal memory	101.3	101.3	0
Executive function	101.2	112.6	11.3
Information processing speed	74.6	80.8	8.3
Attention	87.9	92.1	4.8
Motor skills	104	105.6	1.5

Physical capacity was evaluated by maximal cardiopulmonary exercise test (CPET) conducted on a COSMED treadmill using the Boston 5 protocol. Table 5 presents the pre- and post-HBOT physiological evaluated parameters. As detailed, there was a 34% increase in the VO_2_ max from 3083 to 4130 mL per minute after HBOT. The forced vital capacity (FVC) improved by 44% from 4.76 to 6.87 L, the forced expiratory volume (FEV) by 23% from 3.87 to 4.76 L, and peak flow measurement (PEF) by 20.2% from 10.17 to 12.22 L per second.

**Table 5:** Physiological parameters before and after hyperbaric oxygen therapy

Cardiopulmonary exercise test	Pre-HBOT	Post-HBOT	Change in %
VO_2_ max (mL/min)	3083	4130	34
VO_2max_/kg (mL/min/kg)	31.5	42.4	34.6
Lactic threshold (mL/min)	2941	3439	16.9
Respiratory threshold (mL/min)	3103	4076	31.4
Metabolic equivalent of task (MET)	9	12.1	34.4
Maximal heart rate (bpm)	155	164	5.8
VO_2_/HR (mL per beat)	19.9	25.2	26.6
Pulmonary function tests			
FVC (L)	4.76	6.87	44.3
FEV_1_ (L)	3.87	4.76	23
PEF (L/s)	10.17	12.22	20.2

*VO*_*2*_*max* maximum rate of oxygen consumed during exercise, *ml/min* milliliter per minute, VO_2_max/kg maximum rate of oxygen consumed during exercise per kilogram, *ml/min/Kg* milliliters per minute per kilogram, *MET* metabolic equivalent of task, *bpm* heartbeats per minute, *VO*_*2*_*/HR* rate of oxygen consumed per heart rate, *FVC* forced vital capacity, *L* liters, *FEV1* forced expiratory volume, *PEF* peak flow measurement, *L/s* liters per second

After receiving full information at the end of his post-HBOT evaluation, the patient signed an informed consent allowing publication of his medical information.

## Discussion and conclusions

Here, we report the first case of a patient with long COVID with cognitive and cardiorespiratory symptoms treated successfully by HBOT. Following treatment, he showed significant improvements in brain perfusion, white matter brain microstructure, and cognitive and cardiopulmonary function. This case report shows that HBOT has potential use for treatment of patients with long COVID who suffer from unremitting cognitive and physical functional decline.

Hypoxia plays an important role in the pathophysiology of long COVID. Systemic hypoxia could result from lung impairment, and organ-related hypoxia can develop because of vascular damage. Persisting lung function impairment has been seen in patients who required supplemental oxygen during acute SARS-CoV-2 infection even 6 and 12 months after the acute infection [6]. Since brain functionality and regenerative capacity is sensitive to any decline in oxygen supply [7], long-term cognitive deficits correlate with the amount of oxygen needed to overcome the respiratory difficulties [1]. With regard to organ-related ischemia, COVID-19 induced endothelial damage and hypercoagulation, which increases the risk of vascular dysfunction responsible for the high prevalence of myocardial infarction, ischemic strokes, and pulmonary embolism [8]. In the presented case, the patient required supportive treatment with high-flow oxygen for 1 week during the acute illness, meaning he had suffered from systemic hypoxia with its consequent risk for long-term cognitive impairment due to anoxic brain damage. Moreover, 6 weeks after the acute infection, he developed a pulmonary embolus, representative of the endothelial dysfunction with additional exposure to systemic hypoxia. In addition, as demonstrated by the brain perfusion MRI, he had microvascular-related perfusion defects that correlated with his neurocognitive decline.

HBOT involves the inhalation of 100% oxygen at pressures exceeding 1 atmosphere absolute (ATA), thus enhancing the amount of oxygen dissolved in the body tissues. Even though many of the beneficial effects of HBOT can be explained by improvement of tissue oxygenation, it is now understood that the combined action of hyperoxia and hyperbaric pressure triggers both oxygen- and pressure-sensitive genes, resulting in induction of regenerative processes including stem cell proliferation and mobilization with anti-apoptotic and anti-inflammatory factors, angiogenesis, and neurogenesis [9–12]. HBOT can induce neuroplasticity and improve cognitive function even years after the acute insult [13]. In the case presented of long COVID, HBOT improved cerebral blood flow to the malperfused brain regions (indicative of brain angiogenesis) and improved the integrity of brain microstructure (indicative of neurogenesis). The correlation between the significant improvements demonstrated on brain imaging and the neurocognitive improvements indicates that most of the beneficial effects of HBOT are indeed related to its ability to induce neuroplasticity of the brain’s dysfunctional regions.

HBOT has been demonstrated to have beneficial effects on mitochondrial function, a crucial element of appropriate muscle function [12]. HBOT can also increase the number of proliferating and differentiating satellite cells as well as the number of regenerated muscle fibers, and promote muscle strength [14]. The newly intermittent repeated HBOT protocol was demonstrated to have the potential to improve lung function with respect to peak expiratory flow (PEF) and force vital capacity (FVC) [15]. In the presented patient, performance capacity of the cardiopulmonary system was evaluated using cardiopulmonary exercise test (CPET) and pulmonary function tests. HBOT induced a significant improvement of 34% in the maximal oxygen consumption capacity, an improvement of 34.4% in the maximal METs, and an increase of 16.9% in the lactic threshold. With regard to lung function, FVC was improved by 44.3%, and PEF by 20.2%. These measurable improvements correlated with the patient’s ability to regain his previous high athletic performance.

In this reported case, HBOT was initiated more than 3 months after the acute SARS-CoV-2 infection. Even though the symptoms persisted till the HBOT was initiated and significant improvement began only after HBOT was initiated, it is possible that at least some of the clinical improvement could have occurred without HBOT. However, the abrupt significant improvement with full recovery after the chronic nature of the symptoms, our understanding of the physiological effects of HBOT, and the objective measurements done on this patient support the relation between the treatment and the improvements seen. As this is only a case report, further prospective clinical trials are needed to gain a better understanding of the potential beneficial effects of HBOOT for patients with long COVID.

In summary, this article represents the first case report showing that long COVID can be treated with HBOT. The beneficial effect of HBOT sheds additional light on the pathophysiology of this syndrome. As this is a single case report, further prospective randomized control studies are needed for the use of hyperbaric oxygen therapy in treating long COVID.

## Data Availability

All data generated or analyzed during this study are included in this published article.

## Abbreviations

HBOT: Hyperbaric oxygen therapy
MRI: Magnetic resonance imaging
DTI: Diffusion tensor imaging
VO_2_ max: Maximum rate of oxygen consumed during exercise
CPET: Cardiopulmonary exercise test
HR: Heart rate
Bpm: Heart beats per minute
FVC: Forced vital capacity
FEV_1_: Forced expiratory volume
PEF: Peak flow measurement
